# Pretreatment Peripheral B Cells Are Associated With Tumor Response to Anti-PD-1-Based Immunotherapy

**DOI:** 10.3389/fimmu.2020.563653

**Published:** 2020-10-09

**Authors:** Shumin Yuan, Yuqing Liu, Brian Till, Yongping Song, Zibing Wang

**Affiliations:** ^1^Department of Immunotherapy, Affiliated Cancer Hospital of Zhengzhou University & Henan Cancer Hospital, Zhengzhou, China; ^2^Third Affiliated Hospital of Xinxiang Medical College, Xinxiang, China; ^3^Clinical Research Division, Fred Hutchinson Cancer Research Center, Seattle, WA, United States; ^4^Department of Hematology, Affiliated Cancer Hospital of Zhengzhou University & Henan Cancer Hospital, Zhengzhou, China

**Keywords:** immunotherapy, immune checkpoint inhibitor, PD-1, biomarker, B cells

## Abstract

Identification of reliable biomarkers to predict efficacy of immune checkpoint inhibitors and to monitor relapse in cancer patients receiving this therapy remains one of the main objectives of cancer immunotherapy research. We found that the pretreatment B cell number in the peripheral blood differed significantly between responders and non-responders to anti-PD-1-based immunotherapy. Patients with various cancer types achieving a clinical response had a significantly lower number of B cells compared with those with progressive disease. Patients who progressed from partial response to progressive disease exhibited a gradually increased number of circulating B cells. Our findings suggest that B cells represent a promising biomarker for anti-PD-1-based immunotherapy responses and inhibit the effect of PD-1 blockade immunotherapy. Thus, preemptive strategies targeting B cells may increase the efficacy of PD-1 blockade immunotherapy in patients with solid tumors.

## Introduction

Therapeutic blocking of the programmed death-1 (PD-1) pathway has recently been shown to be a promising strategy for treating solid tumors, rendering long-term survival possible (1). However, only a minority of patients benefit from this treatment. Identification of reliable predictors of response to PD-1 blockade is therefore of utmost importance for determining the most appropriate therapy candidates. PD-L1 overexpression is the most logical biomarker to predict patient response to anti-PD-1 therapy; however, various shortcomings limit its clinical utility, including variability in detection antibodies and differing immunohistochemistry cutoffs (2). Other reports suggest that potential biomarkers could include tumor-infiltrating lymphocytes, mutational burden, and immune gene signatures (3). However, the main obstacle for these biomarkers is the need to obtain tumor biopsies, which is often not feasible, especially for patients with poor performance status or who need to begin therapy urgently. More importantly, even the use of these tumor-based factors fails to reliably identify potentially responsive patients.

Blood is the ideal biological specimen for identifying biomarkers, due to its availability. Hence, analysis of circulating biomarkers as an alternative method is much more convenient for clinical application. Recently, several studies have reported that the proportion of circulating myeloid-derived suppressor cells (MDSCs) differed significantly between responders and non-responders following immunotherapy; patients with a lower frequency of MDSCs had enhanced immune responses when compared with patients who had high MDSC levels (4–8). Furthermore, a recent study found that a set of MDSC-related microRNAs are associated with resistance to treatment with immune checkpoint inhibitors (9). These studies suggest that circulating MDSCs are a potential biomarker for predicting the response to immunotherapy. A recent study investigated the correlation between circulating B cells and clinical response as well as adverse effects in 39 patients with advanced melanoma receiving anti-PD-1 or anti-cytotoxic T lymphocyte-associated protein 4 (CTLA-4) therapy alone, or in combination (10). Although this study did not find a correlation between B cells and clinical response to immunotherapy, it demonstrated that checkpoint blockade immunotherapy led to changes in circulating B cells (10).

In the present study, we set out to identify a reliable biomarker that can be used to predict the response to anti-PD-1-based immunotherapy, and our results strongly suggest that pretreatment levels of B cells are highly predictive of immunotherapy response.

## Materials and Methods

### Study Subjects and Samples

Whole peripheral blood samples from cancer patients were aseptically collected by venipuncture before or after anti-PD-1-based immunotherapy and prepared using a stain-lyse-no-wash procedure to provide white blood cells labeled with fluorescence-linked CD3, CD4, CD8, CD19, CD45, CD16, and CD56 antibodies (BD Multitest 6-color TBNK reagent). Lymphocyte absolute counts and subset percentages are automatically calculated using BD FACSCanto clinical software. The absolute numbers (cells/μL) of positive cells in the sample are determined by comparing cellular events to bead events. The percentages of subsets are obtained by gating the lymphocyte population.

### Statistical Analysis

Data were analyzed using GraphPad Prism Software (GraphPad Software Inc.). Unless otherwise indicated, the results are presented as mean ± standard deviation. The results were analyzed using 2-tailed unpaired or paired Student's *t*-tests. A *p*-value <0.05 was considered significant.

## Results

### Patients and Treatments

A total of 79 patients treated with anti-PD-1-based therapy were included in the study. A detailed listing of patients and treatment characteristics is presented in Table 1. All patients received at least one prior systemic treatment before anti-PD-1-based therapy. Combination immunotherapy approaches involved chemotherapy, radiotherapy, antiangiogenic therapy, and adoptive T-cell therapy. The tumor response was assessed in accordance with the Response Evaluation Criteria In Solid Tumors (RECIST) 1.1. This approach has four response categories: complete response (CR), partial response (PR), stable disease (SD) and progressive disease (PD). Of response-evaluable patients, 31.7% achieved a PR, 22.8% had SD, and 45.6% developed PD, which resulted in a disease control rate of 54.4% (Table 1).

**Table 1:** Characteristics of patients enrolled in this study.

	**Number (*n*, %)**	**BCELL (%)**	**TCELL (%)**	**NKCELL (%)**
**Tumor type**	Malignant melanoma (*n* = 11, 13.92%)	10.05 ± 1.83	68.30 ± 3.04	15.66 ± 2.10
	Lung cancer (*n* = 16, 20.25%)	8.36 ± 0.5	65.79 ± 2.15	21.30 ± 2.44
	Sarcoma (*n* = 8, 10.13%)	7.45 ± 2.36	70.85 ± 2.40	17.1 ± 2.65
	Renal carcinoma (*n* = 12, 15.19%)	7.08 ± 1.78	72.14 ± 2.68	16.72 ± 3.07
	Mammary cancer (*n* = 3, 3.80%)	12.77 ± 1.98	61.63 ± 4.64	19.97 ± 4.04
	Cervical carcinoma (*n* = 5, 6.33%)	21.22 ± 7.49	58.78 ± 10.24	20.78 ± 9.50
	Liver cancer (*n* = 7, 8.86%)	8.81 ± 2.11	66.24 ± 4.07	22.71 ± 4.59
	Lymphoma (*n* = 3, 3.80%)	7.87 ± 5.59	72.17 ± 3.07	17.05 ± 2.38
	Pancreatic cancer (*n* = 2, 2.53%)	4.59 ± 0.35	66.60 ± 8.50	21.05 ± 0.25
	Thymic carcinoma (*n* = 2, 2.53%)	29 ± 26.5	52.40 ± 28.60	12.85 ± 9.15
	Esophageal cancer (*n* = 22.53%)	7.65 ± 1.25	57.20 ± 4.50	26.55 ± 1.65
	Oophoroma (*n* = 1, 1.27%)			
	Oropharynx malignant tumor (*n* = 1, 1.27%)			
	Ulreter carcinoma (*n* = 1, 1.27%)			
	Orchioncus (*n* = 1, 1.27%)			
	Carcinoma tubae (*n* = 1, 1.27%)			
	Gallbladder carcinoma (*n* = 1, 1.27%)			
	Intramedullary glioma (*n* = 1, 1.27%)			
	Vular cystadenocarcinoma (*n* = 1, 1.27%)			
**Gender**	Male (*n* = 42, 53.16%)	7.14 ± 0.87	67.73 ± 1.53	19.20 ± 1.51
	Female (*n* = 37, 46.84%)	13.32 ± 1.83	65.73 ± 2.2	17.52 ± 1.56
**Age (years)**	≤65 (*n* = 66, 83.54%)	11.05 ± 1.18	66.68 ± 1.43	18.94 ± 1.49
	>65 (*n* = 13, 16.46%)	5.65 ± 1.01	67.37 ± 3.32	22.66 ± 2.68
**Clinical response**	PD (*n* = 36, 45.57%)	13.24 ± 1.39	65.09 ± 1.66	17.58 ± 1.67
	SD (*n* = 18, 22.78%)	7.21 ± 1.31	70.05 ± 2.26	18.16 ± 1.92
	PR (*n* = 25, 31.65%)	7.47 ± 2.18	66.92 ± 2.95	21.92 ± 2.36

### Baseline B Cell Number Correlates With Efficacy in Cancer Patients With Anti-PD-1-Based Immunotherapy

**Figure 1:**
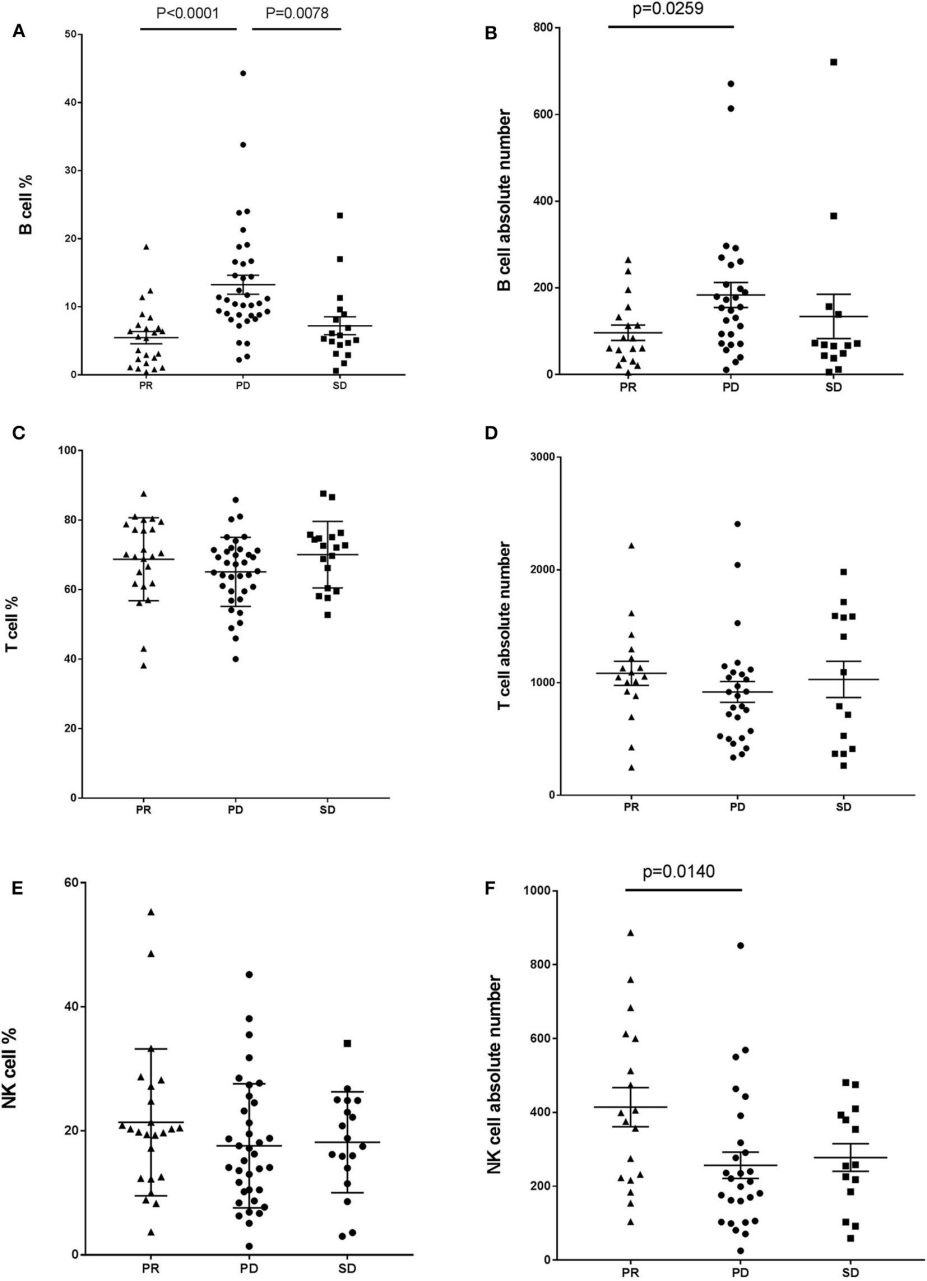
Baseline B cell number correlates with response in cancer patients with anti-PD-1-based immunotherapy. Whole blood cells, obtained from patients before treatment with anti-PD-1-based therapy, were stained and analyzed using flow cytometry. Shown are baseline frequencies, which were expressed as a percentage of total lymphocytes (gated from an CD45 PerCP vs. SSC dot plot) according to clinical response, and absolute number, which were expressed as cells/μL of B cells **(A,B)**, T cells **(C,D)**, and NK cells **(E,F)**. A *t*-test was conducted to test for differences between groups.

We analyzed the absolute lymphocyte counts and lymphocyte subset percentages in the peripheral blood of 78 cancer patients prior to treatment with anti-PD-1-based therapy and evaluated the association with clinical efficacy. A significant difference in the proportion of B cells between responders and non-responders to combination immunotherapy was observed. Patients achieving a PR had a significantly lower frequency of CD19+ B cells compared with that in the patients with PD (Figure 1A). We also found this difference in patients with SD when comparing with patients with PD (Figure 1A). In contrast, no difference was observed in the frequency of CD3+ T cells or CD16+CD56+ natural killer (NK) cells between patients with PD and PR (Figures 1C,E). In addition, no difference was observed in the proportion of T cells or NK cells between patients with PD and SD (Figures 1C,E). Similar results were observed when absolute numbers of lymphocyte subsets were analyzed (Figures 1B,D), except that the value of NK cells was higher in PR patients (Figure 1F). Together, these results indicated that the frequency of B cells in the peripheral blood is associated with efficacy of immunotherapy and might be a potential biomarker for predicting response in patients who receive anti-PD-1-based therapy.

### B Cell Frequency Correlates With Progression in Patients With PR Receiving Anti-PD-1-Based Immunotherapy

**Figure 2:**
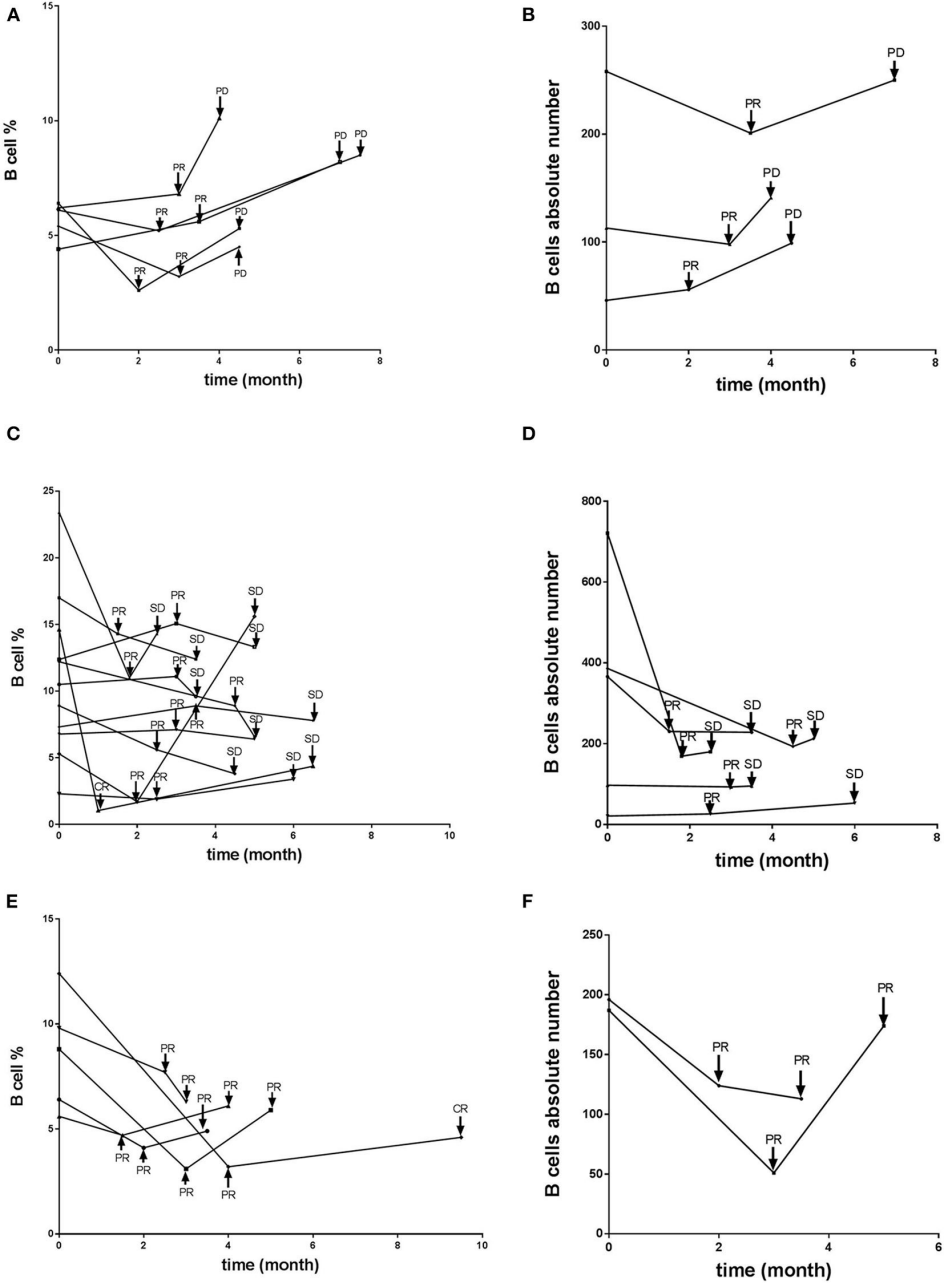
B cell number correlates with progression in patients with PR receiving anti-PD-1-based immunotherapy. Whole blood cells, obtained from patients with PR before and at various timepoints after treatment receiving anti-PD-1-based therapy, were stained and analyzed using flow cytometry. Shown are changes of B cell percentages and absolute numbers in patients with PR who **(A,B)** later developed PD over time, with time of relapse shown with arrows, **(C,D)** maintained stable disease, or **(E,F)** further improved the PR over time.

We next explored whether B cell frequency is able to predict tumor relapse in patients who achieve a PR. Among the 21 patients who achieved a PR, five progressed to PD (PR-PD), 11 exhibited no change (PR-SD), and five had further improvement in their PR over time (PR-PR). B cell counts and imaging studies were performed after every 2–4 cycles of PD-1-based anti-tumor therapy. Most of the patients who progressed from PR to PD exhibited a gradually increased number of circulating B cells (Figures 2A,B), which was not observed in patients who maintained SD or had an improved PR (Figures 2C–F). We also evaluated the association between T cells as well as NK cells and tumor development. No changes were observed whether these patients with PR further improved the PR, maintained SD, or developed PD (data not shown). As of the most recent follow-up, none of the patients in PR-PR or PR-SD groups have relapsed. Together, these data indicate that B cell number might represent a reliable biomarker for monitoring tumor relapse in patients with PR.

### B Cell Absence Leads to Delay of Tumor Growth

**Figure 3:**
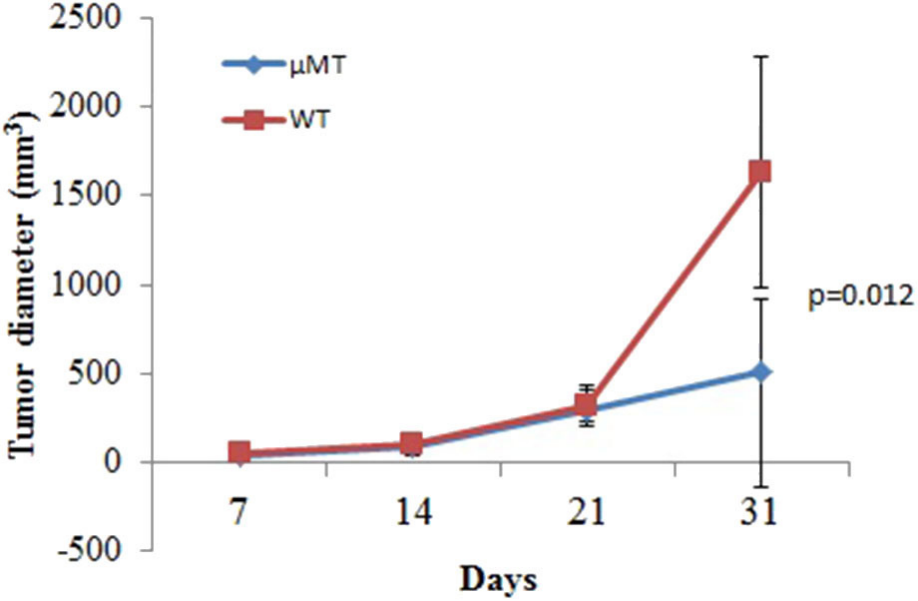
B cell absence leads to delay of tumor growth. WT or μMT mice were injected s.c. with 1 × 105 TSA tumor cells and observed for 31 days (*n* = 5 in each group). Three-dimensional tumor growth was serially measured using calipers and tumor volumes calculated. The average tumor volume (± SD) among all the mice in each group over time is shown.

The aforementioned clinical observation that B cell number is associated with tumor growth suggested that B cell presence might be detrimental to anti-tumor immunity of the host. We tested this hypothesis using a murine model of mammary carcinoma. TSA cells were injected s.c. at day 0 into both syngeneic immunocompetent BALB/c mice as well as μMT mice, which lack B cells but produce normal levels of granulocytes and CD4+ and CD8+ T cells (11). As shown in Figure 3, tumors in the wild-type mice proliferated rapidly. In contrast, the rate of proliferation was significantly reduced in μMT mice. Together, these data suggest that absence of B cells enhances anti-tumor responses.

## Discussion

In the present study, we retrospectively reviewed patients treated with anti-PD-1-based therapy at our center in recent years. We found that patients who developed PD following therapy had a higher number of B cells than those achieving SD and PR, suggesting that B cells in the peripheral blood may impact the efficacy of PD-1-based immunotherapy and might be a potential biomarker for patients who receive this treatment. In addition, an increase in B cell frequency may also be a biomarker identifying patients at high risk for imminent cancer progression after an initial response. These findings need to be confirmed in larger prospective studies enrolling patients with specific cancer types, since this study included a wide variety of cancers.

This study is consistent with our previously published finding that the presence of B cells inhibits induction of T cell-dependent anti-tumor immunity (11). In that study, μMT mice immunized with irradiated TSA cells and challenged 2 weeks later with a lethal dose of the same tumor typically rejected the tumor, whereas nearly all wild-type mice developed a tumor (11). These data suggested, consistent with the present results, that B cells are possibly another negative regulatory factor, in addition to PD-1/PD-L1 co-inhibitory signals, in inducing immune tolerance. PD-1 blockade in combination with B cell inactivation or depletion might be a promising way to achieve more potent responses in tumor immunotherapy.

In conclusion, the data reported herein show that B cells represent a potential predictive biomarker for anti-PD-1-based immunotherapy responses. Preemptive strategies targeting B cells and/or MDSCs may increase the efficacy of PD-1 blockade immunotherapy in patients with solid tumors.

## Data Availability Statement

The raw data supporting the conclusions of this article will be made available by the authors, without undue reservation.

## Ethics Statement

The study protocol was approved by the IRB of Affiliated Cancer Hospital of Zhengzhou University. All participants gave their informed written consent.

## Author Contributions

SY and YL performed experiments, analyzed data, and wrote the manuscript. BT, YS, and ZW analyzed data and edited the manuscript. ZW designed and supervised the study.

## Conflict of Interest

The authors declare that the research was conducted in the absence of any commercial or financial relationships that could be construed as a potential conflict of interest.
