# Supplementary material for: Pretreatment Peripheral B Cells Are Associated With Tumor Response to Anti-PD-1-Based Immunotherapy
Source: Front Immunol. 2020 Oct 9;11:563653. doi: 10.3389/fimmu.2020.563653 (PMC7584071; doi:10.3389/fimmu.2020.563653)
Supplement: Supplementary file 1 [file Table_1.docx]

**Table 1. Characteristics of patients enrolled in this study**

|  |  | **Number** | | **Ratio** | |  |
| --- | --- | --- | --- | --- | --- | --- |
| **Tumor type** | Malignant melanoma | | 11 | | 13.9% | |
|  | Lung cancer | | 16 | | 20.3% | |
|  | Sarcoma | | 8 | | 10.1% | |
|  | Renal cell carcinoma | | 12 | | 15.2% | |
|  | Breast cancer | | 3 | | 3.8% | |
|  | Cervical carcinoma | | 5 | | 6.3% | |
|  | Liver cancer | | 7 | | 8.9% | |
|  | Lymphoma | | 3 | | 3.8% | |
|  | Pancreatic cancer | | 2 | | 2.5% | |
|  | Thymic carcinoma | | 2 | | 2.5% | |
|  | Esophageal cancer | | 2 | | 2.5% | |
|  | Ovarian cancer | | 1 | | 1.3% | |
|  | Oropharynx malignant tumor | | 1 | | 1.3% | |
|  | Ureteral carcinoma | | 1 | | 1.3% | |
|  | Orchioncus | | 1 | | 1.3% | |
|  | Carcinoma tubae | | 1 | | 1.3% | |
|  | Gallbladder carcinoma | | 1 | | 1.3% | |
|  | Intramedullary glioma | | 1 | | 1.3% | |
|  | Vular cystadenocarcinoma | | 1 | | 1.3% | |
| **Gender** | Male | | 42 | | 53.2% | |
|  | Female | | 37 | | 46.8% | |
| **Age (years)** | ≤65 | | 66 | | 83.5% | |
|  | ＞65 | | 13 | | 16.5% | |
| **Response** | PD | | 36 | | 45.6% | |
|  | SD | | 18 | | 22.8% | |
|  | PR | | 25 | | 31.7% | |
| **ECOG PS** | 0  1  2 | | 2  51  26 | | 2.5%  64.6%  32.9% | |
