# Supplementary material for: Pretreatment Peripheral B Cells Are Associated With Tumor Response to Anti-PD-1-Based Immunotherapy
Source: Front Immunol. 2020 Oct 9;11:563653. doi: 10.3389/fimmu.2020.563653 (PMC7584071; doi:10.3389/fimmu.2020.563653)
Supplement: Supplementary file 2 [file Table_2.DOCX]

**Table 2. Concurrent treatments of 79 patients**

| **Treatment** | **Patients with PD**  **(N)** | **Patients with SD**  **(N)** | **Patients with PR**  **(N)** |
| --- | --- | --- | --- |
| **PD-1 monotherapy** | 11 | 4 | 11 |
| **PD-1 plus chemotherapy**  PD-1 plus gemcitabine  PD-1 plus pemetrexed  PD-1 plus liposomal doxorubicin  PD-1 plus pemetrexed/carboplatin  PD-1 plus capecitabine | 5  3  1  1  0 | 1  2  0  0  2 | 4  2  1  0  1 |
| **PD-1 plus adoptive cell transfer**  PD-1 plus CIK cells  PD-1 plus TILs  PD-1 plus TIL plus CIK cells | 9  1  2 | 4  1  0 | 5  0  0 |
| **PD-1 plus targeted drugs** | 1 | 2 | 1 |
| **PD-1 plus chemotherapy plus radiotherapy** | 1 | 0 | 0 |
| **PD-1 plus targeted drugs plus CIK cells** | 1 | 0 | 0 |
| **PD-1 plus radiotherapy plus CIK cells** | 0 | 1 | 0 |
| **PD-1 plus chemotherapy plus CIK cells** | 0 | 1 | 0 |
| **Total** | 36 | 18 | 25 |
