# Supplementary material for: Pretreatment Peripheral B Cells Are Associated With Tumor Response to Anti-PD-1-Based Immunotherapy
Source: Front Immunol. 2020 Oct 9;11:563653. doi: 10.3389/fimmu.2020.563653 (PMC7584071; doi:10.3389/fimmu.2020.563653)
Supplement: Supplementary file 3 [file Table_3.DOCX]

**Table 3. Treatments of patients with PR**

|  | **Patient number** | **Gender** | **Treatment** |
| --- | --- | --- | --- |
| **PR-PD** |  |  |  |
|  | #1 | female | PD-1 plus CIK cells |
|  | #2 | male | PD-1 monotherapy |
|  | #3 | male | PD-1plus TILs |
|  | #4 | female | PD-1 monotherapy |
|  | #5 | male | PD-1 plus targeted drugs |
| **PR-SD** |  |  |  |
|  | #1 | male | PD-1 monotherapy |
|  | #2 | female | PD-1 plus pemetrexed |
|  | #3 | male | PD-1 monotherapy |
|  | #4 | female | PD-1 plus CIK cells |
|  | #5 | female | PD-1 monotherapy |
|  | #6 | male | PD-1 plus CIK cells |
|  | #7 | female | PD-1 monotherapy |
|  | #8 | male | PD-1 plus CIK cells |
|  | #9 | male | PD-1 monotherapy |
|  | #10 | male | PD-1 plus CIK cells |
|  | #11 | male | PD-1 plus gemcitabine |
| **PR-PR** |  |  |  |
|  | #1 | male | PD-1 monotherapy |
|  | #2 | female | PD-1 monotherapy |
|  | #3 | female | PD-1 plus CIK cells |
|  | #4  #5 | female  male | PD-1 plus CIK cells  PD-1 plus CIK cells |
